# Lea’s Series of Medical Epitomes

**Published:** 1911-01

**Authors:** 


**Lea's Series of Medical Epitomes.** Edited by **Victor C. Pedersen,** M.D., New York. **Hygiene and Public Health.** By **George M. Price,** M.D., New York. 12 mo, 255 pages. Lea & Febiger, Publishers, Philadelphia and New York. 1910. (Cloth, $1.00, net.)

Preventive medicine deals with the maintenance of the health of thousands. Curative medicine renders its benefit to individuals singly. This one of the Epitome Series is written by a former medical sanitary inspector of the New York department of health. The aim of the author has been to give the essential facts rather than to cover the entire field, which would be manifestly impossible in a book of this size. Personal hygiene has been entirely omitted from the list of subjects considered, as the author states in the preface. J. A. R.

